# Supplementary material for: Emerging signals of declining forest resilience under climate change
Source: Nature. 2022 Jul 13;608(7923):534–9. doi: 10.1038/s41586-022-04959-9 (PMC9385496; doi:10.1038/s41586-022-04959-9)
Supplement: Supplementary file 1 — This file contains Supplementary Methods 1–3, Discussion 1 and 2, and Figs. 1–3. [file 41586_2022_4959_MOESM1_ESM.pdf]

nature portfolio

https://doi.org/10.1038/s41586-022-04959-9

Supplementary information

Emerging signals of declining forest resilience under climate change

In the format provided by the authors and unedited

Emerging signals of declining forest resilience under climate change

Giovanni Forzieri, Vasilis Dakos, Nate G McDowell, Ramdane Alkama, Alessandro Cescatti

Supplementary Material:

- **Methods 1-3**
- **Discussion 1-2**
- **Figures 1-3**

Methods 1. Thresholds and tipping points

Ecosystems usually respond to gradual change in environmental conditions in a smooth way, however, studies have shown that smooth change can also lead to sudden drastic switches to a contrasting state. Such regime shifts are often related to the crossing of tipping points[12].

A tipping point is a critical threshold in an environmental condition or process that once passed may push the system to shift radically and potentially irreversibly into a different equilibrium state[60]. Supplementary Fig. 1 shows three different theoretical types of linear and nonlinear change in the response of a system in an equilibrium state to changing environmental conditions[12]. The red arrows in the plots indicate the attraction basin, thus the direction in which the system moves if it is not in equilibrium (that is, not on the curve).

The first type of response (Supplementary Fig. 1a) is linear, incremental and gradual following the increasing environmental condition. The change may be reversible along the same trajectory if the environmental condition is decreased or restored to previous levels. A shift in system state can be caused by a sudden large external force. Red arrows indicate that the entire curve represents stable equilibria.

The second type (Supplementary Fig. 1b) shows threshold-dependent, nonlinear behaviour where the system can shift from one state to another. Small perturbations can cause large changes provided that the system is very sensitive in a certain range of conditions. In principle, this change may also be quite easy to reverse. Red arrows indicate that the entire curve represents stable equilibria.

The third type (Supplementary Fig. 1c) also shows threshold-dependent behaviour, but of a special type that mathematically corresponds to a catastrophic fold bifurcation. In this case, there are two stable ecosystem states existing for a wide range of conditions between the two threshold positions F2 and F1. Between F2 and F1 there is an unstable state which represents the border between the basins of attraction of the two alternative stable states on the upper and lower branches (dotted line in Supplementary Fig. 1c). Systems that cross the unstable state or pass the thresholds F1 or F2 will inevitably shift from one to the other stable state.

Methods 2. Relationship between resilience, critical slowing down and early warning signals

The shape and size of the attraction basin determine the stability of a system with multiple stable states. The size of the attraction basin reflects the maximum perturbation that can be taken without causing a shift to an alternative stable state, a property of the system that has been referred in literature as ecological resilience[61]. The slope of the attraction basin (around equilibrium) affects the recovery rate, the capacity of the system to recover from environmental perturbations, typically referred in literature as engineering resilience[62].

In systems with multiple stable states, gradually changing conditions toward a tipping point may have little effect on the state of the ecosystem, but reduce the size and height of the attraction basin ultimately affecting the ability of the system to withstand and recover from environmental perturbations[61,62]. Such loss of resilience makes the system more fragile in the sense that it can easily be tipped into a contrasting state by a small perturbation[12].

When a system approaches a tipping point it may exhibit signs of loss of stability that could potentially be used as early warning signals[5]. In particular, in proximity of a critical threshold, the phenomenon known in dynamical system theory as “critical slowing down” (CSD) may occur[63,64]. At fold bifurcation points (Supplementary Fig. 2a), the dominant eigenvalue characterizing the rates of change around the equilibrium becomes zero. This implies that as the system approaches such bifurcation points, it becomes increasingly slow in recovering from small perturbations[5] and the attraction basin progressively shrinks (Supplementary Fig. 2b,c).

A fundamental implication of CSD is that changes in the recovery rate after perturbations can be used as an indicator of how close a system is to a bifurcation point[64]. Such progressive slowing down leads to an increase in temporal autocorrelation and variance in the resulting pattern of fluctuations[65] (Supplementary Fig. 2d,e). Because slowing down causes the intrinsic rates of change in the system to decrease, the state of the system at any given time becomes more and more like its past state (see box 3 in ref. ([5]) for the mathematical demonstration). A widely used approach to measure the resulting increase in “memory” of the system is to look at lag-1 autocorrelation[53,66], which can be directly interpreted as slowness of recovery in such natural perturbation regimes[64,65].

The fact that CSD may happen across a wide range of complex ecosystems close to tipping points implies a powerful generality for early warning of regime shifts. However, indicators of CSD are not manifested in all cases where regime shifts occur, because not all regime shifts are associated with tipping points[3,4]. Furthermore, recent studies have shown that slowing down generally happens in situations where a system is becoming increasingly sensitive to external perturbations, independently of whether the impeding change is catastrophic or not[14]. This implies that slowing down may be used in a more general sense as a warning signal for a potential decrease in stability.

Methods 3. Forest resilience and disturbance regimes

Forest resilience has been defined as the capacity of an ecosystem to recover after the occurrence of a natural or anthropogenic disturbance event (e.g., wildfires, insect and harvest). Although this interpretation is the classical definition of resilience[67], it is not the only definition used in the literature[68]. Resilience is also defined as the capacity of the system to remain in its current state despite external perturbations (Supplementary Methods 1-2). More importantly, measuring resilience as the rate of recovery does not require a single strong external perturbation, but it can be mathematically equivalent to measuring short-term responses to weak continuous external perturbations[69].

In line with the literature on the topic (e.g., refs. ([3,4,12,15,17–19,53,70–73])), we used the latter concept of resilience in our study. Forest ecosystems are continuously subject to external perturbations (e.g., inter-annual variation of climate drivers), and the capacity of the system to respond to such pressures defines its resilience, which we quantify as changes in temporal autocorrelation. Therefore, resilience in our work is a broader property of forests to withstand perturbations and avoid state shifts, but not the recovery to the initial state after a state change is induced by a major event.

A decline in resilience may increase the susceptibility of forests to abrupt state shifts (or regime shifts) caused by natural disturbances. A sustained degradation of recovery rates following for instance a pro-longed rise in temperature can make the forests more vulnerable to insect outbreaks[25]. However, not all natural disturbances are associated with a loss in resilience. Indeed, many disturbances represent fast and abrupt shock that are not anticipated by a change in resilience, so they may occur regardless on the changes in resilience[74]. For example, fires are typically not driven by ecosystem resilience, but on the contrary from specific climatic conditions and the occurrence of an ignition. Additional details on what regime shifts are announced by a decline in resilience are reported in ref. ([4]). Therefore, declining forest resilience should not be interpreted as a direct indicator of increasing forest disturbances but instead they should be viewed as emergent signals of an increasing instability of forests.

Discussion 1. Emergent relationships between long-term TAC and environmental drivers

We used partial dependence plots (PDPs) derived from the machine-learning algorithm Random Forest (RF) to explore the marginal effect of a set of environmental drivers on the long-term TAC and gain insights on the potential underlying ecological mechanisms responsible of the changes in forest resilience (Methods). We found that low values of long-term TAC typically occur at locations characterised by humid climates and high forest-cover density (Extended Data Fig. 2b,c). This suggests that, at the global level, favourable growing conditions and high recovery rates overlap with structural and compositional diversity[32,33]. The analysis shows that the background climate has a clear influence on recovery rates (Extended Data Fig. 2b,d). For instance, water limitations slow down the ability of forests to recover from disturbances and reduce regeneration[46]. Low recovery rates (high values in long-term TAC) are observed for temperatures above 12°C and precipitation levels below 1500 mm, consistently with previous studies[15,16,75] (Extended Data Fig. 1b and Extended Data Fig. 2b,d). In addition to aridity, high climate variability appears linked to low recovery rates (Extended Data Fig. 2b,e). Indeed, worldwide cases of widespread tree mortality have been associated systematically to extreme events, such as droughts and heatwaves[76]. Furthermore, we found that autocorrelation in climate forcings represent a primary driver of long-term TAC (Extended Data Fig. 2b,f), thus suggesting a strong interplay between the temporal dynamic of climate and vegetation.

Discussion 2. Results of the sensitivity analysis

Sensitivity to the quality flag. The NDVI quality flag (QF) determines the reliability of the original satellite retrievals and therefore affects the robustness of the derived estimates of forest resilience. The quality flags "good" (description: "use with confidence") and "marginal" (description: "useful, but look at other QA information") are typically those flags utilized for remote sensing applications. In general, estimates based exclusively on the good quality flag are more robust but have lower spatial and temporal coverage compared to those derivable including also data with marginal quality flags. We tested two different quality screening: QF = good and QF = good & marginal.

Changes in the quality flag (QF) do not affect the frequency distributions of the differences in TAC at the global scale and in tropical, temperate and boreal climate regions (Extended Data Fig. 4a,b,d,e). Differences emerge in arid forests with a percentage of forest area affected by a decline in resilience slightly higher when considering both good and marginal quality flags compared to the experiment including only the good quality flag (63% vs. 57%, Extended Data Fig. 4c). However, both screening experiments confirm a dominant decline in arid forests and show an overall high consistency in the patterns of the differences in TAC in the climate space (Extended Data Fig. 5a-b).

Sensitivity to the gap filling. The gap filling procedure (GF) aims to reconstruct missing data in the NDVI time series to allow the computation of the temporal autocorrelation by assuring the integrity of the time series. To this scope, missing data have been gap-filled by the climatological kNDVI values after de-seasonalization and de-trending of the original time series. Such approach implicitly assumes that the anomaly in the kNDVI signal is zero for the specific element in the time series that is gap-filled. This assumption seems plausible for the large majority of missing data occurring in the boreal region and that are caused by the limitation in kNDVI retrievals due to high snow and cloud cover during the boreal winter. In order to verify the robustness of such approach we tested the differences in TAC using kNDVI time series retrieved for different period of the year. The first experiment includes all kNDVI estimates retrieved over the year (GF=year), while the second experiment uses only kNDVI values over the climatological growing season (GF=growing season) derived from the Vegetation Index and Phenology (VIP) satellite-based product[54] (see Methods). Therefore, the number of gap-filled values included in this second experiment is negligible compared to the first experiment and therefore it can be assimilated to an experiment without gap filling.

Results show that the gap filling (GF) of off-season kNDVI records minimally affect the frequency distributions of the differences in TAC at the global scale and in each single climate region with slightly lower kurtosis when TAC values are computed only for growing season periods compared to analogous estimates based on the annual periods (Extended Data Fig. 4f-j). Overall, there are no substantial changes in terms of percentage of forest area affected by a decline in resilience and the gap filling experiments show an overall high consistency in the patterns of the differences in TAC in the climate space (Extended Data Fig. 5c-d).

Sensitivity to the inclusion/exclusion of areas affected by abrupt declines. The definition of resilience we follow throughout the manuscript is the one that reflects the capacity of the system to remain close to its expected steady state when perturbed. We measure this capacity in temporal changes of TAC. Abrupt declines (AD) in the vegetation state and following recoveries, as those potentially originating from major forest disturbances (e.g., wildfires, insect outbreaks), can influence autocorrelation and potentially introduce spurious trends in TAC (negative or positive) depending on the spatial and temporal distribution of such disturbances in the time series under investigation. For instance, in the first years of recovery from a strong perturbation vegetation typically shows lower fluctuations, and thus lower TAC compared to the pre-disturbance conditions. In order to assess if the inclusion/exclusion of these events is affecting the results of the analysis we tested two contrasting conditions: all areas affected by abrupt declines are included in the analysis (AD=incl.), all areas affected by abrupt declines are excluded (AD=excl.). Pixels affected by AD occurring over the 2010-2020 period are identified based on the methodology described in the Methods (section "Early warning signals of abrupt forest declines", with abrupt declines of 3σ severity). The analysis was complemented with the identification of AD events occurring over the 2000-2009 period by replicating the same methodology after a flipping of the kNDVI time series along the time dimension.

Results show that the inclusion or exclusion of forest areas affected by abrupt declines does not affect substantially the frequency distribution of the temporal differences in TAC at the global scale and at climate region level (Extended Data Fig. 4k-o). Overall, a very minor amplification (~1%) of the increase in TAC can be observed when areas affected by AD are excluded from the analysis. We conclude that AD occurrences, being distributed throughout the whole period under analysis, do not introduce a bias in the assessment of the temporal changes in TAC because of compensatory effects. This is further corroborated by the high consistency in the patterns of the differences in TAC in the climate space amongst the two AD experiments (Extended Data Fig. 5e-f).

Sensitivity to the percentage of missing data. The percentage of missing data (PMD) allowed at the pixel scale influences the spatial domain of analysis. Pixels with PMD above a fixed threshold are masked out and are excluded from the analyses of forest resilience. Lower values of PMD lead to a smaller spatial domain but characterized by pixels with a higher number of NDVI retrievals, the opposite holds for higher values of PMD. We tested three different PMD thresholds: PMD<20%, PMD<50% and PMD<70%.

Changes in PMD do not affect the frequency distribution of the differences in TAC at the global scale and in tropical, arid and temperate climate regions (Extended Data Fig. 4p,q,r,s), as these zones show a very limited number of missing data. More evident effects manifest in boreal forests because imagery is frequently affected by snow cover conditions causing poor NDVI quality retrievals (Extended Data Fig. 4t). The kurtosis of the distribution reduces markedly with increasing PMD values. Furthermore, we note that in the experiment with a reduced number of missing data allowed (PMD<20%) there emerges a slightly larger fraction of boreal forests experiencing a decline in resilience compared to those subject to an opposite trend in resilience (52% vs. 48%). This signal is consistent with that observed in the other climate regions but

appears of opposite sign with respect to that found in boreal forests under the two experiments with a higher number of missing data allowed (PMD<50% and PMD<80%). This results from the change in spatial domain of boreal forests. Indeed, more stringent conditions in PMD lead to a reduction of boreal forest domains particularly in cold-dry climate conditions (e.g., Canada and European Russia) where we observe large areas with increasing trends in resilience. Regardless of the overall signal obtained by aggregating the data over different spatial domains, the patterns of the difference in TAC (2000-2010 and 2011-2020) emerge consistent in the climate space across all PMD-based domains showing an increasing trend in resilience in colder and dryer zones and an opposite signal in milder and wetter zones (Extended Data Fig. 5g-i).

Sensitivity to the percentage of forest cover. The percentage of forest cover (PFC) allowed at the pixel scale influences the spatial domain of the analysis, similarly to PMD. Pixels with PFC below a fixed threshold are masked out and are excluded from the analyses of forest resilience. Higher values of PFC lead to smaller spatial domain but characterized by pixels more representative of the forest conditions (higher forest extents at pixel level), the opposite holds for lower values of PFC. We tested three different PFC thresholds: PFC>5%, PFC>50% and PFC>90%.

Changes in PFC minimally affect the frequency distribution of the differences in TAC at the global scale and in tropical and boreal climate regions (Extended Data Fig. 4u,v,y), as more extensive forest covers compared to the rest of the globe typically characterize these zones. Differences are more evident in arid and temperate climate regions (Extended Data Fig. 4w,x) where forests show a high level of fragmentation. In these regions, we note that the frequency distributions shift to higher positive values with increasing PFC value. In temperate regions 63% of the area shows a decline in resilience for PFC>5%, while 73% for PFC>90%. Arid regions show similar patterns: 57% and 61% of the area show a decline in resilience for PFC>5% and PFC>90%, respectively. This suggests that in these regions, the observed decline in resilience tends to manifest more markedly in areas characterized by extensive forests where the contamination effects of non-forest areas are more limited. This amplification effect is also reflected in the patterns of the differences in TAC in the climate space computed for different PFC domains (Extended Data Fig. 5j-l).

Sensitivity to the pixel spatial resolution. The pixel spatial resolution (PSR), determining the level of spatial aggregation of the signal, influences the capacity of our modelling framework to capture the spatial and temporal variations in forest resilience. We tested three different PSRs: PSR=0.05°, 0.25° and 1°.

Increasing PSR values induce a systematic change in the frequency distributions of the differences in TAC, as reflected by a shift towards higher positive values in all climate regions (Extended Data Fig. 4z-ad). Therefore, coarser spatial resolutions lead to an amplification of the dominant signal of decreasing resilience. This effect appears prominent in tropical forests where 56% of the area shows a decline in resilience for PSR=0.05°, while 65% for PSR=1°. The observed amplification presumably results from a reduction of potential spatial noise, which allows the detection of more marked temporal variations in forest resilience at coarser spatial resolutions, at the expenses of a reduced capacity to capture fine-scale processes. Such emerging

signals are confirmed in the patterns of the differences in TAC in the climate space computed for different PSR domains (Extended Data Fig. 5m-o).

Sensitivity to the lagged temporal window length. The length of the lagged temporal window (TWL) determines the number of records used to retrieve the annual TAC value and the resulting length of the TAC time series. Shorter temporal windows tend to provide less robust estimates of TAC, whereas longer temporal windows reduce the length of the time series to be analyzed. We tested three different TWLs: TWL=1 year, 2 years and 3 years.

The trend in total TAC obtained from the three experiments did not show substantial differences across the climate gradients (Extended Data Fig. 6a-c). There was a consistent decline of resilience in tropical, arid and temperate climate regions and an opposite signal in boreal zones, with changes of increasing magnitude for larger TWLs. Overall, the forest areas with declining TAC (enhanced resilience) tended to slightly increase with larger TWL, particularly in cold-dry climates. Larger TWLs probably mitigate the detrimental effects of the higher number of missing data in these regions and plausibly contribute to improve the estimate of TAC.

Overall assessment. Results of the sensitivity analysis shown in Extended Data Figs. 4-6 and discussed above corroborate the robustness of our findings with respect to the modelling choices adopted in our approach (Methods). Therefore, the implemented model setup (QA=good and marginal, GF=year; AD=incl.; PMD<50%, PFC>5%, PSR=0.05° and TWL=3 years) appears a reasonable compromise to properly capture the spatio-temporal dynamics of forest resilience.

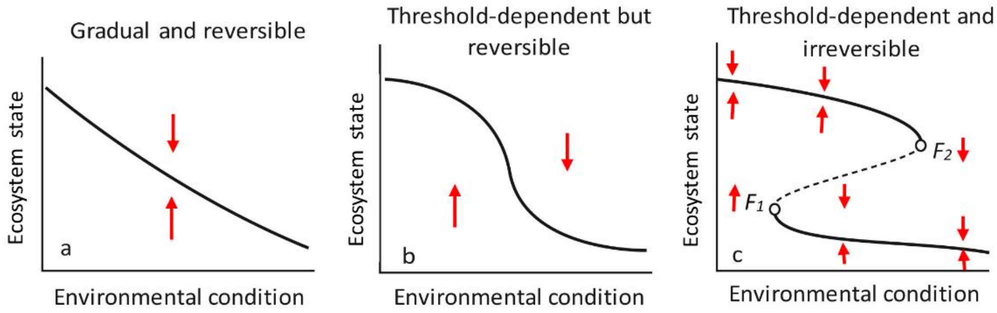

Figure 1. System change in response to a range of conditions. (**a**) Gradual and reversible behaviour. (**b**) Threshold-dependent but reversible behaviour. (**c**) Threshold-dependent and irreversible behaviour from a catastrophic fold bifurcation. Figure modified from ref. ([12]).

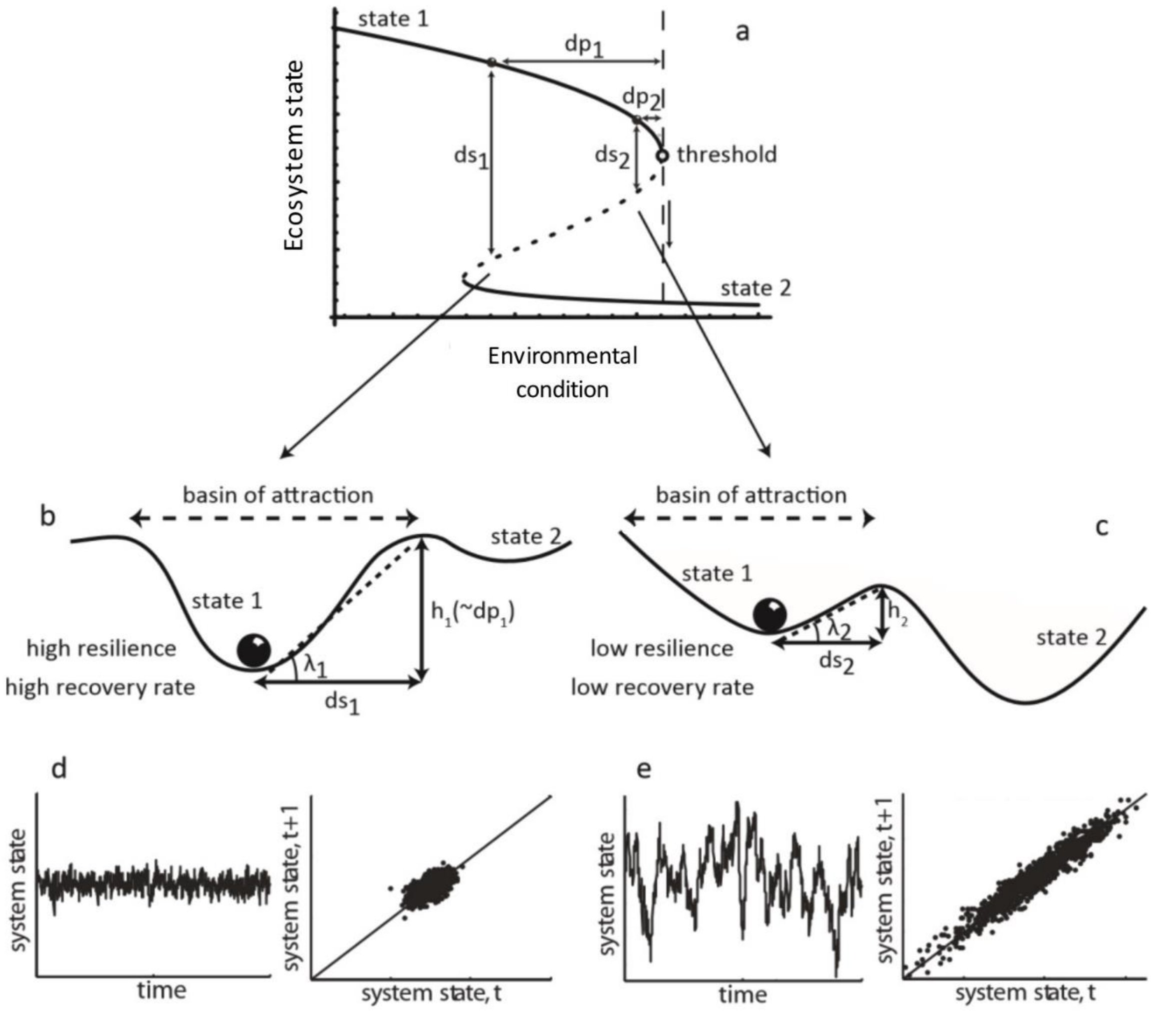

Figure 2. Changes in non-equilibrium dynamics as a system approaches a catastrophic bifurcation. (**a**) Threshold-dependent transition due to a catastrophic fold bifurcation. (**b**,**d**) Far from the bifurcation point, resilience is large in two respects: the basin of attraction is large and the rate of recovery form perturbations is relatively high. When the system is perturbed, the resulting dynamics are characterized by low correlation between states at subsequent time intervals. (**c**,**e**) Close to the bifurcation point resilience decreases in two senses: the basin of attraction shrinks and the rate of recovery from small perturbations is lower. Following such slowing down, the system shows a longer memory and its dynamics are characterized by a larger variance and a stronger correlation between subsequent states. ds_x is the distance to the unstable manifold (dotted line in (**a**)) that reflects the maximum amount of disturbance the system can tolerate without shifting states, dp_x is the distance to the fold bifurcation in parameter space (environmental condition), h_x is the depth of the basin of attraction, λ_x is the slope of the basin of attraction (the dominant eigenvalue estimated locally at equilibrium). Figure modified from ref. ([4]).

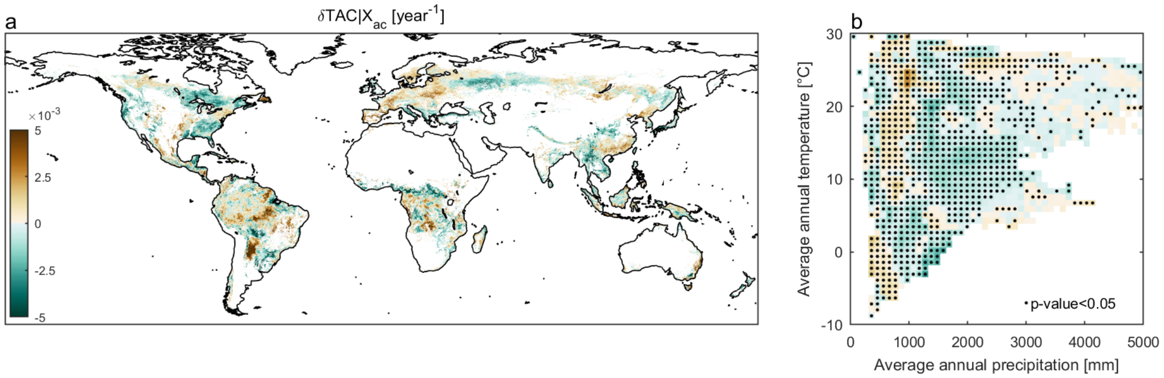

Figure 3. Temporal variations in forest resilience due to autocorrelation in climate forcings. (**a**) Spatial map of trend in forest resilience due to autocorrelation in climate ($\delta TAC|X_{ac}$). (**b**) $\delta TAC|X_{ac}$ binned as a function of climatological precipitation and temperature. Black dots indicate bins with average values that are statistically different from zero (two-sided Student's *t*-test; P-value ≤ 0.05).
