# Supplementary material for: Emerging signals of declining forest resilience under climate change
Source: Nature. 2022 Jul 13;608(7923):534–9. doi: 10.1038/s41586-022-04959-9 (PMC9385496; doi:10.1038/s41586-022-04959-9)
Supplement: Supplementary file 2 — Reporting Summary [file 41586_2022_4959_MOESM2_ESM.pdf]

nature portfolio

Corresponding author(s): Giovanni Forzieri

Last updated by author(s): Apr 19, 2022

Reporting Summary

Nature Portfolio wishes to improve the reproducibility of the work that we publish. This form provides structure for consistency and transparency in reporting. For further information on Nature Portfolio policies, see our Editorial Policies and the Editorial Policy Checklist.

Statistics

For all statistical analyses, confirm that the following items are present in the figure legend, table legend, main text, or Methods section.

n/a	Confirmed	
☒	☐	The exact sample size (*n*) for each experimental group/condition, given as a discrete number and unit of measurement
☒	☐	A statement on whether measurements were taken from distinct samples or whether the same sample was measured repeatedly
☐	☒	The statistical test(s) used AND whether they are one- or two-sided<br>*Only common tests should be described solely by name; describe more complex techniques in the Methods section.*
☐	☒	A description of all covariates tested
☒	☐	A description of any assumptions or corrections, such as tests of normality and adjustment for multiple comparisons
☐	☒	A full description of the statistical parameters including central tendency (e.g. means) or other basic estimates (e.g. regression coefficient) AND variation (e.g. standard deviation) or associated estimates of uncertainty (e.g. confidence intervals)
☐	☒	For null hypothesis testing, the test statistic (e.g. *F*, *t*, *r*) with confidence intervals, effect sizes, degrees of freedom and *P* value noted<br>*Give P values as exact values whenever suitable.*
☒	☐	For Bayesian analysis, information on the choice of priors and Markov chain Monte Carlo settings
☒	☐	For hierarchical and complex designs, identification of the appropriate level for tests and full reporting of outcomes
☒	☐	Estimates of effect sizes (e.g. Cohen's *d*, Pearson's *r*), indicating how they were calculated

Our web collection on statistics for biologists contains articles on many of the points above.

Software and code

Policy information about availability of computer code

Data collection	MATLAB 2017R
Data analysis	MATLAB 2017R. The custom MATLAB (R2017b) code written to analyse the data, develop the random forest model and generate figures is available at https://doi.org/10.6084/m9.figshare.19636059.v1.

For manuscripts utilizing custom algorithms or software that are central to the research but not yet described in published literature, software must be made available to editors and reviewers. We strongly encourage code deposition in a community repository (e.g. GitHub). See the Nature Portfolio guidelines for submitting code & software for further information.

Data

Policy information about availability of data

All manuscripts must include a data availability statement. This statement should provide the following information, where applicable:

- Accession codes, unique identifiers, or web links for publicly available datasets
- A description of any restrictions on data availability
- For clinical datasets or third party data, please ensure that the statement adheres to our policy

The climate datasets used in this study are publicly available from the ERA5-land reanalysis product (https://cds.climate.copernicus.eu/cdsapp#!/home) and from Köppen-Geiger World map of climate classification (http://koeppen-geiger.vu-wien.ac.at/present.htm). Normalized Difference Vegetation Index (NDVI) data are acquired from the Moderate Resolution Imaging Spectroradiometer (MOD13C1 Version 6, https://lpdaac.usgs.gov/products/mod13c1v006/), land surface phenology data from the Vegetation Index and Phenology (VIP) satellite-based product (https://vip.arizona.edu/) and forest cover data from the European Space Agency's Climate Change Initiative (ESA-CCI, https://www.esa-landcover-cci.org/). Gross Primary Productivity fluxes are available from the FLUXCOM product (http://www.fluxcom.org/) and the spatial delineation of intact forests from the Intact Forest Landscapes dataset (http://intactforests.org/).

Field-specific reporting

Please select the one below that is the best fit for your research. If you are not sure, read the appropriate sections before making your selection.

☐ Life sciences ☐ Behavioural & social sciences ☒ Ecological, evolutionary & environmental sciences

For a reference copy of the document with all sections, see nature.com/documents/nr-reporting-summary-flat.pdf

Ecological, evolutionary & environmental sciences study design

All studies must disclose on these points even when the disclosure is negative.

Study description	Our contribution provides the first observation-based global-scale assessment of how forest resilience evolved in recent decades in response to global change. For this purpose, we developed a novel methodology that integrates theoretical bases of the resilience of nonlinear dynamical systems approaching a tipping point, satellite-based kernel Normalized Difference Vegetation Index, recently proposed as a strong proxy for ecosystem productivity, and machine learning techniques.
Research sample	Spatial and temporal variations in forest resilience were retrieved from MODIS NDVI data, analysed at the pixel scale (0.05°) and aggregated per climate regions (globe, tropical, arid, temperate, boreal) and forest types (managed, intact). Analyses produced at the pixel level were meant to explore the local variations in forest resilience. Analyses conducted separately per climate region and forest type were aimed at characterizing the overall trajectories of forest resilience over large climatically consistent zones and disentangling the human-induced effect on vegetation dynamics (e.g., forest management). The MODIS NDVI product used in this study (MOD13C1 Version 6) being derived from a unique platform and sensor, it is temporally and spatially consistent. Furthermore, the 16-day acquisition time interval and the available temporal coverage (2000-2020) make the MOD13C1 product a suitable dataset to explore spatial and temporal variations in forest resilience.
Sampling strategy	When statistics are analyzed at climate region/forest type scale, we included in the sample all pixels that have passed the screening procedure (see section below “Data exclusions”). Sample sizes are typically larger than 50000 pixels (Supplementary Table 2) and therefore are considered fully representative of the climate region/forest type investigated.<br>When results are binned in a 50x50 grid as a function of annual precipitation and temperature (e.g., Fig. 1b), we retained only bins with at least 50 records. Such sampling strategy appears a reasonable compromise to identify major climate features and in parallel to reduce possible noise in bins poorly representative.<br>When differences between managed and intact forests are analyzed, the potential effect of climate background has been removed. To this aim, we compared the climate spaces generated separately for managed and intact forests by extracting only those bins that are covered by both forest classes. The resulting distributions - one for each forest class - have the same sample size and each pair of elements shares the same climate background. This method allows to filter out the potential confounding effect of climate background in the two classes of forests. Similar approach was used to calculate the probability of abrupt decline conditional on negative trend in resilience. The resolution of the climate spaces (50x50 bins) ensures samples of sufficient sizes for statistical analyses.
Data collection	All data utilized in this study are acquired from satellite-based datasets and reanalysis products (see section “Data availability”).
Timing and spatial scale	We quantify the spatial patterns of forest resilience at the global scale and explore its temporal evolution over the period 2000-2020.
Data exclusions	Resilience indicators were derived for forest pixels with less than 50% missing data in the original NDVI data of good and marginal quality and with forest cover greater than 0.05. In order to assess the robustness of our results with respect to the modelling choices described above we performed a series of sensitivity analyses.<br>Sensitivity to the quality flag. The NDVI quality flag (QF) determines the reliability of the original satellite retrievals and therefore affects the robustness of the derived estimates of forest resilience. The quality flags “good” (description: “use with confidence”) and “marginal” (description: “useful, but look at other QA information”) are typical quality flags utilized for remote sensing applications. In general, estimates based exclusively on the good quality flag are more robust but have lower spatial and temporal coverage compared to those derivable including also data with marginal quality flags. We tested two different quality screening: QF = good and QF = good & marginal.<br>Sensitivity to the percentage of missing data. The percentage of missing data (PMD) allowed at the pixel scale influences the spatial domain of analysis. Pixels with PMD above a fixed threshold are masked out and are excluded from the analyses of forest resilience. Lower values of PMD lead to a smaller spatial domain but characterized by pixels with a higher number of NDVI retrievals, the opposite holds for higher values of PMD. We tested three different PMD thresholds: PMD<20%, PMD<50% and PMD<70%.<br>Sensitivity to the percentage of forest cover. The percentage of forest cover (PFC) allowed at the pixel scale influences the spatial domain of the analysis, similarly to PMD. Pixels with PFC below a fixed threshold are masked out and are excluded from the analyses of forest resilience. Higher values of PFC lead to smaller spatial domain but characterized by pixels more representative of the forest conditions (higher forest extents at pixel level), the opposite holds for lower values of PFC. We tested three different PFC thresholds: PFC>5%, PFC>50% and PFC>90%.<br>Overall assessment. Results of the sensitivity analysis shown in Extended Data Figs. 4-6 and presented in Supplementary Discussion 2 corroborate the robustness of our findings with respect to the modelling choices adopted in our approach (Methods). Therefore, the implemented model setup (QA=good and marginal, PMD<50%, PFC>5%) appears a reasonable compromise to properly capture the spatio-temporal dynamics of forest resilience.
Reproducibility	Our data-driven modelling is highly reproducible within the computational accuracy on other computing platforms.
Randomization	The Random Forest regression model (RF) we have developed is based on:<br>1) random record selection: each tree is built from a separate random sample of the data using bootstrap sampling.

2) random predictor selection: in a standard tree, each split is created after examining every predictor and selecting the best split from the number of predictors to sample.
The RF implemented in our study uses 100 regression trees, whose depth and number of predictors to sample at each node were identified using Bayesian optimization.

Blinding: n/a

Did the study involve field work? ☐ Yes ☒ No

Reporting for specific materials, systems and methods

We require information from authors about some types of materials, experimental systems and methods used in many studies. Here, indicate whether each material, system or method listed is relevant to your study. If you are not sure if a list item applies to your research, read the appropriate section before selecting a response.

Materials & experimental systems

n/a	Involved in the study	
☒	☐	Antibodies
☒	☐	Eukaryotic cell lines
☒	☐	Palaeontology and archaeology
☒	☐	Animals and other organisms
☒	☐	Human research participants
☒	☐	Clinical data
☒	☐	Dual use research of concern

Methods

n/a	Involved in the study	
☒	☐	ChIP-seq
☒	☐	Flow cytometry
☒	☐	MRI-based neuroimaging
